# Antibody-Mediated Rejection in Lung Transplantation: Diagnosis and Therapeutic Armamentarium in a 21st Century Perspective

**DOI:** 10.3389/ti.2024.12973

**Published:** 2024-08-07

**Authors:** Jonathan Messika, Natalia Belousova, François Parquin, Antoine Roux

**Affiliations:** ^1^ Thoracic Intensive Care Unit, Foch Hospital, Suresnes, France; ^2^ Physiopathology and Epidemiology of Respiratory Diseases, UMR1152 INSERM and Université de Paris, Paris, France; ^3^ Paris Transplant Group, Paris, France; ^4^ Pneumology, Adult Cystic Fibrosis Center and Lung Transplantation Department, Foch Hospital, Suresnes, France; ^5^ Université Paris-Saclay, INRAE, UVSQ, VIM, Jouy-en-Josas, France

**Keywords:** lung transplantation, antibody-mediated rejection, diagnosis, diagnosis biomarker, therapeutic approaches

## Abstract

Humoral immunity is a major waypoint towards chronic allograft dysfunction in lung transplantation (LT) recipients. Though allo-immunization and antibody-mediated rejection (AMR) are well-known entities, some diagnostic gaps need to be addressed. Morphological analysis could be enhanced by digital pathology and artificial intelligence-based companion tools. Graft transcriptomics can help to identify graft failure phenotypes or endotypes. Donor-derived cell free DNA is being evaluated for graft-loss risk stratification and tailored surveillance. Preventative therapies should be tailored according to risk. The donor pool can be enlarged for candidates with HLA sensitization, with strategies combining plasma exchange, intravenous immunoglobulin and immune cell depletion, or with emerging or innovative therapies such as imlifidase or immunoadsorption. In cases of insufficient pre-transplant desensitization, the effects of antibodies on the allograft can be prevented by targeting the complement cascade, although evidence for this strategy in LT is limited. In LT recipients with a humoral response, strategies are combined, including depletion of immune cells (plasmapheresis or immunoadsorption), inhibition of immune pathways, or modulation of the inflammatory cascade, which can be achieved with photopheresis. Altogether, these innovative techniques offer promising perspectives for LT recipients and shape the 21st century’s armamentarium against AMR.

## Introduction

Humoral immunity has been found to be a major waypoint towards acute and chronic lung allograft dysfunction (CLAD) [1–3], with the human leukocyte antigen (HLA) system being the tip of the iceberg. Alloimmunization and subsequent antibody-mediated rejection (AMR) are now well-known entities in solid-organ transplantation. Nevertheless, although the knowledge around AMR has been growing during the last two decades, several questions still arise in the clinical field of lung transplantation (LT). Difficulties in AMR diagnosis, which cannot rely solely on donor-specific antibody testing, are a significant challenge in LT, where the diagnosis of definite, clinical AMR is relatively rare event [4–6]. The presence of circulating antibody in the absence of clinical graft dysfunction may be of no importance - or signify chronic, smoldering AMR that ultimately accelerates the onset of chronic lung allograft dysfunction and graft loss despite the absence of an acute syndrome. The biggest challenge in AMR diagnosis is to differentiate between these two types of apparently subclinical events.

Other challenges include treatment of alloimmunization in the pre-transplant period, in order to expand the donor pool for highly sensitized candidates. After transplantation, therapeutic challenges include the initiation of pre-emptive treatment of AMR in at-risk recipients, the actual treatment of clinical AMR and, in-between, the opportunity to treat subclinical AMR. These strategies have caveats, and the existing armamentarium is being enriched by therapies currently under investigation.

## AMR in Lung Transplantation: From Myth to Reality

The earliest description of a harmful effect from antibodies directed against a graft was published in 1969 [7], describing increased frequency of immediate graft failure in kidney transplants with a positive crossmatch. The first case attributing hyperacute rejection in lung transplant to donor-specific anti-HLA antibodies (DSA) was published in 1996 [8]. However, the existence of AMR as a clinical entity in LT remained a matter of some debate even into the early 21st century [9]. A series of publications in the 2000s [10–13] describing histological patterns associated with HLA antibodies, and both acute and chronic graft dysfunction, contributed to the growing recognition of LT AMR, but the 2007 revision of lung rejection diagnosis and nomenclature [14] held back from making recommendations on AMR diagnosis. The authors cited insufficient knowledge, at the time of publication, of the true sensitivity and specificity of different diagnostic tools [14].

A multidimensional consensus statement aimed at standardizing AMR diagnosis was finally published in 2016 [15], and recommended diagnosis based on a combination of circulating DSA, evidence of complement activation, histological pattern, graft dysfunction and exclusion of other diagnoses, such as acute cellular rejection (ACR) or infection. The recommendations included three levels of diagnostic certainty (definite, probable and possible AMR) as well as definitions for clinical and sub-clinical AMR. The consensus statement allows a thorough classification and is the best tool currently available. A revision of these guidelines is ongoing and should be available in the next few months. However, several diagnostic gaps still need to be addressed. Emerging diagnostic strategies and novel molecular and digital techniques can improve the precision of AMR diagnosis and classification.

## Addressing Diagnostic Gaps of AMR in Lung Transplantation

### Graft Dysfunction

Graft dysfunction is a major component of the diagnosis of clinical AMR in LT in the current guidelines. However, few studies clearly describe how graft dysfunction is diagnosed. As this is the sole feature distinguishing clinical and subclinical AMR [15] and it has prognostic implications [6], a clear definition is essential.

The ISHLT consensus statement defines allograft dysfunction as “alterations in pulmonary physiology, gas exchange properties, radiologic features or deteriorating functional performance” [15]. This description leaves much room for interpretation. Broadly, a 10% decline in the forced expiratory volume in 1 s (FEV_1_) is frequently quoted as a threshold to intervene/investigate, but does this really indicate “graft dysfunction”? And how rapidly should the drop have occurred – intuitively, it seems that a 10% drop over 5 days has different implications to the same drop observed over a year. Furthermore, in the first 6–12 months post-transplant, when FEV_1_ values are expected to improve until the patient achieves their baseline, even a stable FEV_1_, as opposed to an increasing one, can be indicative of underlying abnormalities and graft damage. On the other hand, acute respiratory distress syndrome is obviously considered as graft dysfunction, even though the patient is unlikely to be able to perform pulmonary function testing in this context. Further studies should evaluate different dimensions of graft dysfunction and how these dimensions affect clinical outcome.

One potential avenue is enhanced assessment of CT images with the aid of quantitative image analysis and machine learning. The current AMR guidelines do not mention radiology, and its value currently lies in the assessment for other causes of acute graft dysfunction, such as infection or pulmonary edema, as well as guiding transbronchial biopsy. ACR and AMR may both present with ground-glass opacities, pleural effusions and interlobular septal thickening [16, 17]. However, machine learning techniques have already been shown to be capable of differentiating different phenotypes of CLAD and predict graft outcome [18, 19]. It is possible that a similar approach, applied in an acute setting, could help to differentiate different causes of acute graft dysfunction, including AMR.

### Anti HLA Antibodies

Anti-HLA antibodies directed against the donor (DSA) have been associated with both acute and chronic rejection in kidney and heart transplantation. HLA antibodies are detected by single antigen bead (SAB) assays, in which latex beads coated with specific HLA antigens are mixed with the patient’s serum, and a secondary, fluorochorome-conjugated anti-human IgG antibody, is used to generate a fluorescent signal whose intensity corresponds to the amount of HLA-specific antibody bound to each bead. The mean fluorescence intensity (MFI) is what is usually reported to the clinician and used to guide management decisions; however, it is at best a semi-quantitative measure of circulating antibody concentration, with a measurement variability that may reach 25% [20]. Standardized cutoff values for positivity have not been established [21, 22]. Antibody binding sites, or epitopes, on each bead may be bound by more than one antibody, therefore it is possible to saturate the beads, and produce a high MFI even though the actual amount of any specific antibody present in the serum is relatively low; on the other hand, a single epitope might be present on several beads (shared epitope), therefore reducing the MFI at the bead level. The antibody titer is a much more accurate measure of antibody concentration and is associated with graft outcomes.

Other antibody characteristics to consider include its specificity and complement activation capacity [23–26]. Class II DSA, particularly those against the DQ locus, carry a higher risk of CLAD and mortality compared to Class I DSA, or non-donor-specific HLA antibodies [2, 4]. Risk stratification according to DSA specificity should be considered, in order to guide (or refrain from) intervention. It should be noted that while research tends to focus on DSA, pre-transplant sensitization even with non-donor specific antibodies carries an increased risk of developing *de novo* DSA after transplant [27]. Older studies show an overall association between the presence of anti-HLA antibodies and shorter graft survival [3, 28, 29], though improved immunosuppression techniques, approaches to the sensitized transplant candidate and the ability to identify DSA have blunted this overall effect in the modern transplant era [3].

Patients can exhibit high titers of DSA without demonstrating any clinical graft dysfunction, while other patients can demonstrate graft dysfunction and other features of AMR without any detectable circulating DSA [30]. The existence of a clinical syndrome suggestive of AMR in the absence of circulating DSA is still debated, and explanations are speculative: missing donor HLA in the single antigen kit, intragraft DSA production with adsorption [31], epitope sharing [32], DSA targeting non HLA epitopes [33], and IgM DSA [25] are among the possibilities raised in DSA-negative cases otherwise suspicious for AMR.

### Non-HLA DSA

The implication of non-HLA antibodies as the trigger for AMR has emerged in some reports [34–36], as it has been suspected in kidney transplantation [37]. Non-HLA DSA include non-HLA alloantigen, with more than 1,000 possible targets [37]; or autoantigen (e.g., collagen V, collagen I, and K-α 1 Tubulin, endothelin, AT1R) [34, 35]. If non-HLA DSA was previously considered to be a simple graft injury bystander, their harmful effect has been demonstrated by Xu et al. [33]. In this retrospective study, an increased risk of CLAD was observed in patients who had non-HLA DSA; moreover, a synergic effect was noted for patients who had both HLA- and non-HLA DSA [33].

### Complement Activation

The clinical relevance of DSA may also depend on its capacity to bind complement. C1q binding has been cited as a potential adjunct to determine the significance of a circulating DSA, however, it is not one of the diagnostic criteria in the 2016 ISHLT consensus statement [15]. C1q activation requires the presence of 6 antibody molecules in close proximity and is thus a consequence of high concentrations of DSA [20]. C1q fixation has been associated with shorter time to CLAD and reduced graft survival [24], but this is not consistently demonstrated across studies [38]. More recently, Roux et al. [23] demonstrated an association between C3d activation and poorer graft survival rates. Moreover, a strong C3d activation was associated with higher DSA titers.

Tissue complement activation is detectable with positive C4d staining on the transbronchial biopsy (TBB). However, C4d staining can be associated with other conditions (infection, ACR, diffuse alveolar damage), and should therefore always be interpreted in the clinical context [15]. Conversely, negative C4d staining may reflect technical limitations rather than the absence of complement activation: C4d deposition in alveolar capillaries may be patchy or granular (and therefore interpreted as negative); interobserver agreement between different pathologists and different staining techniques has also been shown to be suboptimal [39, 40]. Moreover, a variety of non-complement dependent mechanisms of AMR have been described [41].

Aguilar et al demonstrated that even in C4d-negative AMR, 67% of patients had C1q positivity, suggesting that C1q and C4d act as complementary biomarkers of complement involvement in a given AMR process. However, no survival difference was observed between patients with C4d positive and C4d negative AMR, reinforcing the idea that C4d staining is not a necessary criterion for AMR diagnosis [39]. The added value of TBB C4d staining or circulating DSA associated C3d and C1q activation may be primarily in the selection of therapeutic strategies [42–44].

### Histological Features of AMR

Histological features on TBB that have been identified to be suggestive of AMR include neutrophilic capillaritis, neutrophilic septal margination, organizing pneumonia and high-grade ACR [15]. Aside from the overlap with ACR or another concurrent diagnosis (Table 1), these features suffer from additional challenges in their role as diagnostic markers, including poor interobserver reliability [45, 46] and difficulty in identifying the compartmentalization of neutrophils in capillaries. Furthermore, reporting practices vary from center to center, which is a particular challenge for retrospective studies. Efforts such as the Lung Allograft Standardized Histological Analysis (LASHA) [47], are underway to standardize reporting practices.

**TABLE 1:** Histological patterns suggestive of antibody-mediated rejection and its associated differential diagnosis.

Histological pattern	Differential diagnosis
Neutrophilic margination	Infection Ischemia-reperfusion injury (in a compatible timeline)
Neutrophilic capillaritis	Infection Ischemia-reperfusion injury (in a compatible timeline)
Acute lung injury pattern/diffuse alveolar damage	Infection Toxic inhalation Ischemia-reperfusion injury (in a compatible timeline)
Persistent/recurrent acute cellular rejection (any A grade)	Persistent/recurrent acute cellular rejection without AMR component
High-grade acute cellular rejection (≥A3)	High-grade acute cellular rejection without AMR component Infection
Persistent low-grade lymphocytic bronchiolitis (grade B1R)	Infection Gastroesophageal reflux disease Low-grade lymphocytic bronchiolitis without AMR component
High-grade lymphocytic bronchiolitis (grade B2R)	Infection Gastroesophageal reflux disease High-grade lymphocytic bronchiolitis without AMR component
Obliterative bronchiolitis (grade C1)	Chronic rejection
Arteritis	Infection Acute cellular rejection without AMR component
Any histologic findings in setting of DSA positivity (e.g., AFOP)	Infection

All diagnosis should be considered with consideration of clinical presentation, results of other investigations (bronchoalveolar lavage microbiology, DSA…) and response to therapies.

AFOP, acute fibrinous and organizing pneumonia; AMR, antibody-mediated rejection; BAL, bronchoalveolar lavage; DSA, donor specific antibody.

The availability of high-throughput digital pathology slide scanners and advances in computing hardware, network spends and artificial intelligence (AI) tools, now allow the processing of large quantities of image data and have opened new avenues for AI to improve the accuracy and reproducibility of histopathological assessment. The success of such methods has been demonstrated in the oncology field, where deep learning algorithms were able to identify tumor features on pathology slides, and were associated with patient outcomes in lung cancer [48]; furthermore, they have even been shown to out-perform pathologists in detection of lymph node metastases on whole-slide images [49]. Studies are underway in heart, kidney and liver transplant [50]. In LT, the Duke University group [51] used AI to assess digitalized TBB slides annotated by LT pathologists to identify areas of normal lung tissue and A1/A2 grade ACR lesions. The algorithm was able to distinguish the vascular component of ACR with 95% accuracy. It is hoped that similar methods can be applied to the diagnosis of AMR to assist in identification of AMR-associated lesions.

Novel staining approaches are also being investigated. For instance, the phosphorylated S6 ribosomal protein was found to have a higher expression in TBB sampling of patients with AMR compared with controls [52]. These promising results should be confirmed in prospective studies.

## Emerging Diagnostic Strategies

### Transcriptomic Analysis

Transcriptomic analysis is increasingly being utilized in kidney and heart transplantation, in conjunction with a thorough clinical contextualization, to identify patterns of gene expression associated with rejection phenotypes. In LT settings, Halloran et al. [53] used microarray technology in TBB sampling, and identified archetypes associated with ACR, but not with AMR. The microarray approach has limitations, such as the need forof a dedicated lung sample, which is therefore unavailable for histological analysis, or the lack of accurate clinical contextualization, preventing the inclusion of transcriptomic results in a multidimensional approach. Transcriptomic analysis with alternative techniques, such as nanostring technology [54, 55], is under investigation for the diagnosis of AMR and other rejection phenotypes.

### Donor-Derived Cell-Free DNA

Donor-derived cell-free DNA (dd-cfDNA) has recently gained a lot of attention as a marker of graft injury. Higher levels of dd-cfDNA have been observed in patients with clinical dysfunction associated with various injuries, including AMR compared to patients with stable function. Furthermore, a rise in dd-cfDNA preceded diagnosis of clinical AMR, but not ACR, by 2.8 months [5]. Another prospective multicentric cohort study reported a negative predictive value to rule out AMR or ACR ranging from 90% to 96% [56]. Although promising, these results deserve further validation. Keller et al. reported an independent association of dd-cfDNA level with CLAD or death. Unfortunately, the analysis suffers from a relatively small numbers of patients and the lack of assessment of the clinical severity of rejection episode, precluding any definitive conclusion on clinical utility in real life settings [57]. In our opinion, the lack of clinical contextualization would jeopardize this promising tool. Moreover, the severity of graft dysfunction should not rely on a biomarker but on clinical assessment.

## Existing Armamentarium for Desensitization, Alloimmunization, and AMR

**FIGURE 1:**
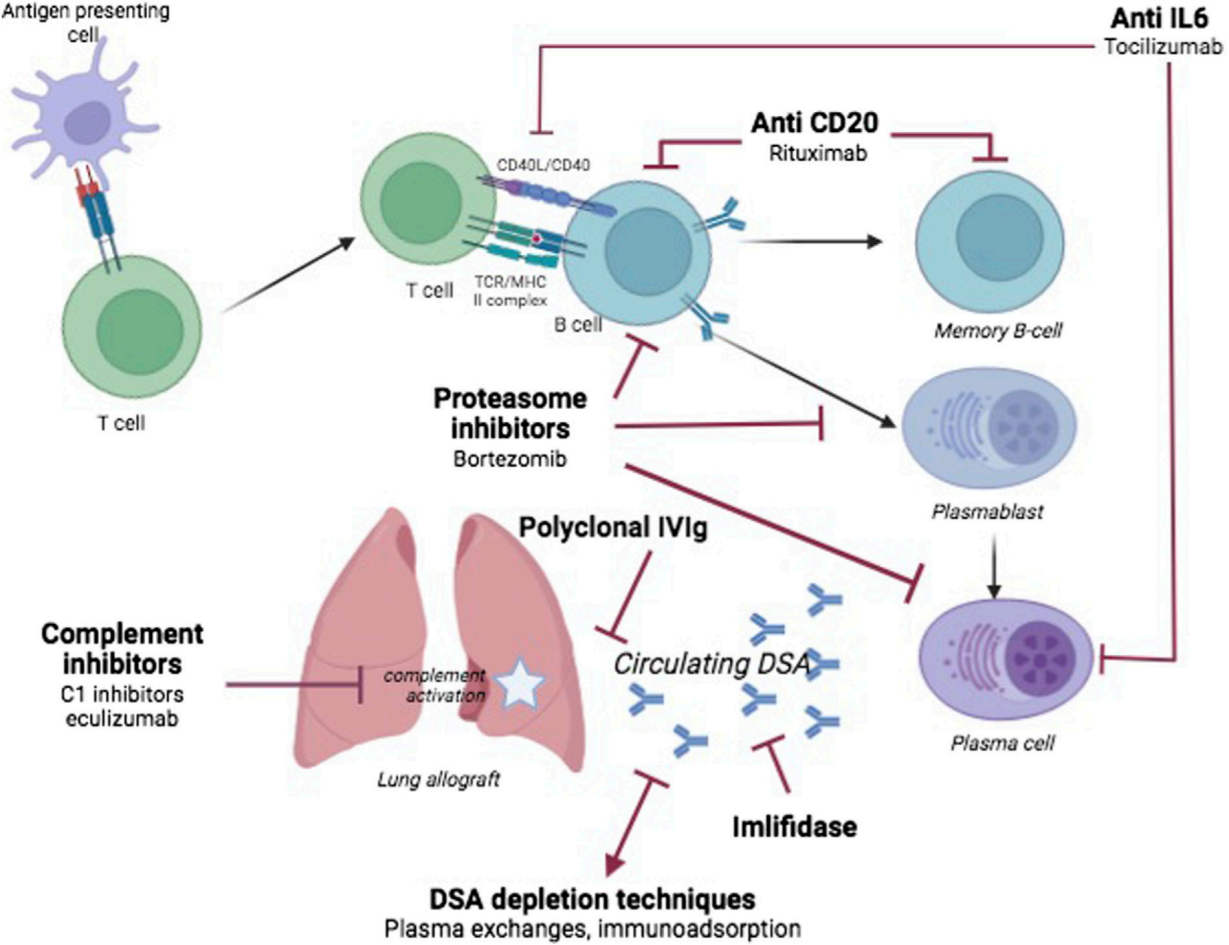
Schematic overview of antibody-mediated rejection in lung transplantation. The main therapies discussed are depicted with their targets.

Anti-HLA antibodies can be found at all stages of the LT process and can impact the patient and the graft, on the waitlist or after transplantation. DSA is known to be associated with all forms of rejection, whether it be hyperacute [8], acute or chronic [10–13], thus leading to graft loss [1, 2, 58]. The identification of situations with risks for developing *de novo* DSA, and the calibration of the therapeutic response, are therefore vital in order to prevent—or limit—CLAD occurrence and evolution. Each clinical step corresponds to a specific scenario, but a shared armamentarium is used. A representation of AMR pathophysiology and the treatments discussed are depicted in Figure 1.

The preventive step aims at overcoming preexisting immunization in a candidate. In lung allografts, conflicting data have been reported with regards to the detrimental effects of pre-transplant sensitization. Several studies report an impaired prognosis of LT recipients with a history of DSA [1, 2, 8, 11–13, 28, 58] but others did not identify any detrimental effect of pre-transplant sensitization [59] provided adequate screening is performed [60]. Regardless of the conflicting data, allosensitized candidates have a decreased likelihood of transplant compared with non-allosensitized, and higher odds of death on the waiting list [51, 52], with a significant association between elevated calculated panel-reactive antibody (cPRA), and decreased likelihood of transplant. In these cases, treatments administered before or during surgery aim to desensitize the recipient to anti-HLA antibodies and prevent the detrimental effects of pre-formed DSA at the time of transplantation [61–67]. A virtual crossmatch strategy based on historical immunization helps to mitigate the risks associated with the allocation of an allograft to a sensitized recipient. In our center, a virtual cross-match is systematically performed with the historical pre-formed DSAs detected on the date of listing. In most cases, allocation of a proposed graft to an immunized candidate with DSAs is forbidden if MFI is over 5,000. If the historical DSA MFI falls between 500 and 5,000, transplantation is allowed, but a peri-operative a desensitization protocol is applied [62].

After LT, the emergence of DSA in the absence of any clinical or histopathological pattern of AMR is still an issue, and the question of whether it should trigger early treatment or close monitoring remains unanswered.

The decision to initiate AMR treatment mandates the assessment of the clinical severity of the episode, the type of DSA and its epitope specificity, its titer (or mean fluorescence intensity - MFI), and the presence of possible complement activation. Table 2 is a suggested proposal for a therapeutic approach to alloimmunization and AMR. Of note, it reflects only our center’s approach, integrating bibliography, expert opinions, local experience, clinical constraints of specific patients, and logistic constraints of facilities. It is therefore open to discussion, including within our own team, and should be considered with caution, tailored to each patient, and adapted to the circumstances of each center and HLA laboratory.

**TABLE 2:** Therapeutic approaches to alloimmunization and antibody-mediated rejection including subclinical and clinical forms, with or without complement activation.

Mechanism and therapy	Indications	Schedule and duration	Effect onset	Side-effects
DSA Clearance				
Plasma exchanges Immunoadsoprtion	Alloimmunization with high MFI and anti-DQ Clinical AMR with or without complement activation	Plasma exchanges: daily, 5–7 days	Few hours	
Imlifidase	Pre-LT desensitization Rescue for clinical AMR with or without complement activation	Once	Few hours	Infection; transaminitis; Anaphylaxis, serum sickness
DSA production inhibition				
Anti CD20	Alloimmunization Subclinical or clinical AMR, with or without complement activation	375 mg/m^2^ twice at D0 and D7 to D30; can be repeated at M6	72 h for B-cell depletion; weeks to months for antibody decrease	Anaphylaxis; neutropenia; hypogammaglobulinemia; infection
Proteasome inhibitors – Bortezomib	After anti CD20, in case of persisting DSA and subclinical or clinical AMR, with or without complement activation	1.3 mg/m^2^, divided in 4 infusions between D1 and D11	Few hours for plasma cell depletion; weeks to months for antibody decrease	Peripheral neuropathy; neutropenia
Neutralization of intra graft DSA				
IVIG	Subclinical or clinical AMR, with or without complement activation	2 g/kg monthly for 6 months	Few hours	Renal impairment; hypervolemia; hyperviscosity
Complement inhibitors	Clinical AMR, with complement activation	C1-esterase inhibitor: 20 IU/kg twice weekly for 6 months	Few hours	Encapsulated infectious counterparts

Currently available treatments can target each step of the humoral response, usually in combination: antibody removal, inhibition of antibody production, inhibition of DSA effect and immunomodulation.

It has to be underlined that existing evidence does not allow to draw definitive conclusions: different strategies and associations are reported, at different time-points of the LT recipient course, and in various situations of moving definitions. We therefore aim to list this existing armamentarium, and potential areas for future research.

### Antibody Removal

Antibody removal is a cornerstone of the treatment of humoral response for both desensitization and AMR treatment. It can be achieved by plasmapheresis or immunoadsoption.

During plasmapheresis, plasma with its protein components is removed, and replaced by colloids, albumin, or fresh frozen plasma. Plasma exchange removes anti-HLA antibodies; but as it is a non-specific therapy, it also removes other large proteins, including coagulation factors, and anti-infectious immunoglobulins. Replacement by colloids, albumin, or fresh frozen plasma is essential to avoid coagulopathy [68]. In LT, the experience reported by Snyder et al. [64] is disappointing. They report the outcome of 18 highly sensitized candidates who underwent a desensitization protocol, including plasmapheresis, bortezomib, rituximab, and IVIg, among whom 9 received a LT. Their prognosis did not differ significantly than their non-desensitized alloimunised counterparts. The experience of the Toronto team, in a similar peri-operative situation, is more optimistic, and reported in both short-(65) and long-term [63] perspectives. Their strategy includes an assessment of the immunological risk, and a combination of plasma exchanges, IVIg, and thymoglobulin. Among 340 patients in their cohort, 53 had DSA. Four did not undergo any treatment. All of the remaining received plasma exchange, 43 received IVIg, and 23 of those received thymoglobulin. Interestingly, the DSA-positive patients were less likely to develop grade 2 ACR, similarly to those who had a cPRA above 30%, but no DSA. Respiratory function, 30-day survival and 1-year survival did not differ between groups (DSA-positive, cPRA>30% or neither) [65]. They extended the follow-up of these patients for a median of 6.7 years, and showed no differences in graft survival, CLAD-free survival, or overall survival [63]. In a study by the Foch Lung Transplant group [62], a perioperative desensitization protocol was applied in all recipients who had preformed DSA with MFI between 500 and 5,000. The protocol included one pre-operative plasma exchange followed by 5 plasma exchange sessions starting on postoperative day-1, a rituximab infusion, and finally 2 g/kg intravenous immunoglobulins. The mycophenolate mofetil dosage for maintenance immunosuppression was also increased if the MFI of the preformed DSA was above 1,000 on day 0. In this series, the 39 patients who had been desensitized because of high preformed DSA were compared to the 66 who had low preformed DSA, and the 216 who were not pre-sensitized. The outcome did not differ according to the presence of preformed DSA, in terms of freedom from CLAD, or 1 and 3-year graft survival. In contrast, these outcomes differed significantly according to successful clearance of the DSA. These data support an aggressive strategy of preformed DSA clearance in order to improve long-term outcomes.

Immunoadsorption has been developed in order to specifically remove IgG. As with plasma exchange, the plasma is separated from the whole blood, but instead of being discarded, it circulates through a column coated with a protein that binds the fixed region of immunoglobulins. Only the immunoglobulin antibodies are adsorbed, while other circulating proteins are reinjected into the patient, allowing a massive decrease in total IgG. Various immunoadsorption devices have been developed over time, in order to refine the removed proteins. Some use immobilized antibodies and deplete all subclasses of IgG. Others use immobilized staphylococcal protein A, and deplete IgG autoantibodies, and circulating immune complexes containing IgG. Moreover, these devices are thought to carry beneficial immunosuppressive effects via B-cell apoptosis. Finally, some columns might carry immobilized antigens or synthetic epitopes in order to only extract the antibodies that are reactive with a single antigen [69]. The Duke University LT program team reported its experience with a desensitization regimen including IVIg and extracorporeal immunoadsorption [70]. In this center, during an 11-year period, 12 patients who had anti-HLA antibodies at the time of transplantation were desensitized, while 23 were not. Patients who underwent desensitization had fewer episodes of acute rejection and higher (although non-significant) freedom from BOS in the first 3 years. These data support the efficacy of a strategy which encompasses extracorporeal immunoadsorption, but, to our knowledge, no comparison between different antibody removal techniques has been performed.

### Inhibition of DSA Effects

The use of intravenous immunoglobulins (IVIg) was first reported in kidney transplant [71, 72]. They are believed to neutralize the existing antibodies, blocking the effect of DSA on the allograft, and to downregulate B-cells. To date, most protocols reported in LT, whether it be for desensitization [64, 65], or treatment of alloimmunization with or without AMR, include IVIg in combination with other therapies [1, 58, 73–76].

### Inhibition of Antibody Production: Administration of B-Lymphocyte or Plasma-Cell Depletion Treatments

Most of existing protocols consider the use of B-lymphocyte or plasma-cell depletion treatments [1, 58, 73–76], such as rituximab (anti-CD20 antibody) and bortezomib or carfilzomib (proteasome inhibitors), to be mandatory. Their administration aims at inhibiting the production of DSA and is a complementary step to DSA removal. Rituximab is widely used, either for desensitization [62], in alloimmunization [58, 73, 75] and AMR [1, 75]. It has been combined with bortezomib [64, 75, 76] or thymoglobulin [65]. One has to bear in mind the delay of action of rituximab, which depletes B cells within 72 h, but does not affect plasma cells or existing antibody levels. The effect on DSA is therefore seen only after a few months [77, 78]. Thymoglobulin, on the other hand, depletes B cells, T cells, NK cells and terminally-differentiated plasma cells, exerting a more immediate effect on antibody production [79, 80]. Proteasome inhibitors induce apoptosis of plasma cells, via the accumulation of ubiquitinated proteins. For instance, carfilzomib acts within 1 h of first administration and is thought to inhibit proteasome function for >48 h after each dose.

## Emerging Therapies

Other strategies are being used in case reports or small series. The following strategies are still being scrutinized and not routinely used in LT at the time of writing.

### Imlifidase

Imlifidase is an IgG-degrading enzyme derived from *Streptoccous pyogenes*. It inactivates IgG antibodies by cleaving their lower hinge region. It has been successfully tested in highly immunized kidney transplant candidates [81], in spite of the risk of a secondary antibody rebound between day 3 and day 14 [81]; moreover, it has been reported to have an excellent post-transplant prognosis [82]. Such results had even led to a French consensus report on hypersensitized kidney transplant candidates [83], positioning imlifidase as an alternative to apheresis. In the LT literature, a single case has been published [66]. In this highly immunized candidate, LT was made possible after a dramatic decrease of anti HLA antibodies secondary to imlifidase administration and followed by an aggressive desensitization strategy with C1 esterase inhibitor, plasma exchange, alemtuzumab and IVIg.

### Targeting of the Complement Cascade

The complement cascade is suspected to be an important pathway of AMR induced lung injury [15, 55], as it is in kidney [84], liver, and heart transplantation. Anti-complement drugs have therefore been used, in order to mitigate the local inflammatory response and thus the local effects of AMR. They are expected to be effective in cases of AMR with evidence of circulating complement-activating anti-HLA DSA [44].

*Eculizumab,* an anti-C5 monoclonal antibody, has been reported to be effective in various settings. For instance, in AMR occurring in the early course of LT, it has been used in combination with a more conventional strategy of AMR treatment [85, 86]. Both cases report the successful treatment of AMR in a LT recipient, either with hyperacute AMR on post-operative day-2, combined with bortezomib, rituximab, IVIg, and plasma exchange [85]; or acute AMR, occurring on post-operative day 7, and successfully treated with a combination of eculizumab, IVIg and rituximab [86]. Highly sensitized kidney [87] and heart [88] transplant recipients treated with eculizumab have been found to have a better prognosis than their non-treated counterparts. The integration of eculizumab in a desensitization strategy in highly sensitized candidates is an interesting possibility that should be investigated.

*C1-esterase specific inhibitors* are still being scrutinized in AMR, mostly in kidney recipients [42]. Data are scarce in LT. After being investigated in the very early course of LT in order to limit primary graft dysfunction [89, 90], the use of C1-esterase inhibitors has been reported in 2 LT recipients with AMR refractory to standard of care [43], with successful treatment. Both patients had acute respiratory failure, with DSAs, and histology pattern consistent with AMR. While one of the patients had very early respiratory failure (on post-operative day-2), the other had respiratory failure 3 years after LT. Both patients received the treatment for a prolonged course of 6 and 7 months respectively. The first patient improved, and the deterioration of the second plateaued, stabilizing the patient and allowing retransplantation. In both cases, IVIg was maintained along with C1-esterase inhibitors. No adverse effect has been described in these two cases. In spite of this encouraging case report, and of a strong pathophysiological rationale to use this therapeutic strategy, the research on C1-esterase inhibitors in pulmonary AMR remains sparse: to date, no trial is registered in the ClinicalTrials.gov database.

### Immunomodulation by IL-6 Inhibitors

The use of the IL-6 inhibitor tocilizumab for AMR in LT is a matter of debate. Tocilizumab is a potent anti-inflammatory treatment and has been reported to treat kidney AMR [91, 92]. It has been reported in a single retrospective case series in LT [93]: the authors compared the outcome of 18 LT recipients who were diagnosed with AMR (Definite, n = 5; Probable, n = 12; Possible n = 1) receiving combination therapies, with 9 LT recipients with AMR (Definite, n = 2; Probable, n = 7) whose combination included tocilizumab. The results are encouraging, albeit non-significant: tocilizumab recipients had more DSA clearance, less DSA recurrence and less development of new DSA. Interestingly, whereas lung function did not differ either at AMR diagnosis or at follow-up, graft failure was significantly lower in the patients receiving tocilizumab. While this interesting paper has some limitations, it nevertheless provides valuable data, that could pave the way for a prospective trial. Clazakizumab is another IL-6 inhibitor, which is being investigated in a phase 3 trial in chronic AMR in kidney transplant recipients (NCT 03744910). To date, no trial in LT has been reported.

### Extracorporeal Immunomodulation by External Chemo-Phototherapy (ECP)

ECP is a therapy used in various situations including in solid organ transplantation [94]. In LT, it has been mainly investigated in chronic lung allograft dysfunction [95–104]. It is based on the principle of isolation of white blood cells into an extracorporeal circuit, their sensitization to ultraviolet radiation by a photoactivable material (8-methoxypsoralen), and, after UV-A exposure, reinjection into the patient’s circulation. It is thought to promote induction of lymphocyte apoptosis and production of T regulatory cells. A single paper by the Vienna team describes the use of ECP in AMR [105]. In this single center retrospective study, ECP was used as an add-on therapy in 16 of 41 LT recipients with AMR. The first-line treatment was immunoadsorption in 14 of these 16 patients, ATG + IVIg in one patient and ATG alone in one patient. Two of the immunoadsorption patients also received IVIg, and 2 others received ATG. The authors report a reduction of *de novo* DSA titers, a 1-year survival of 55% and a 1-year graft survival rate of 61%. This study provides encouraging results and demands ongoing investigation of this strategy. The study EXPORT-DSA, led by the Vienna LT team, is registered in the ClinicalTrials.gov database (NCT06112951). It is a prospective randomized trial of ECP in patients with persistent *de novo* DSA, without any sign of graft dysfunction. This study is not recruiting at the moment but should provide valuable insights on possibility of reducing DSA with ECP treatment.

## Conclusion

As the pathophysiology of antibody-mediated rejection is better and better understood, the unmet needs in diagnosis and treatment progressively shrink. Several unanswered questions in AMR diagnosis may be addressed with the help of big data and novel diagnostic strategies. While there remains a great deal of heterogeneity in approaches to alloimmunization and AMR treatment, a tailored phenotypic characterization would allow a multimodal therapeutic approach, with innovative techniques and treatments, some of which are already in use in other organ transplantation fields. They provide promising perspectives for LT recipients and shape the 21st century’s armamentarium against AMR.

## Group Members of The Foch-Lung Transplant Group

Laurence Beaumont, Olivier Brugiere, Francesco Cassiano, Charles Cerf, Johanna Cohen, David Cortier, Sylvie Colin de Verdiere, Jerome Devaquet, Julien De Wolf, Virginie Dumans, Julien Fessler, Michael Finet, Richard Galliot, Matthieu Glorion, Sindia Goncalves, Louis Grosz, Amer Hamdan, Abdul-Momen Hamid, Charles Hickel, Morgan Le Guen, Sebastien Jacqmin, Julien Josserand, Nicolas Mayenco-Cardenal, Chloe Mimbimi, Sandra de Miranda, Alexis Paternot, Mathilde Phillips-Houlbracq, Clement Picard, Ciprian Pricopi, Julien Richard, Edouard Sage, Guillaume Tachon, Benjamin Zuber.
